# M-current modulation of cortical slow oscillations: Network dynamics and computational modeling

**DOI:** 10.1371/journal.pcbi.1011246

**Published:** 2023-07-05

**Authors:** Leonardo Dalla Porta, Almudena Barbero-Castillo, Jose Manuel Sanchez-Sanchez, Maria V. Sanchez-Vives

**Affiliations:** 1 Institut d’Investigacions Biomèdiques August Pi i Sunyer (IDIBAPS), Barcelona, Spain; 2 ICREA, Passeig Lluís Companys, Barcelona, Spain; Inria, FRANCE

## Abstract

The slow oscillation is a synchronized network activity expressed by the cortical network in slow wave sleep and under anesthesia. Waking up requires a transition from this synchronized brain state to a desynchronized one. Cholinergic innervation is critical for the transition from slow-wave-sleep to wakefulness, and muscarinic action is largely exerted through the muscarinic-sensitive potassium current (M-current) block. We investigated the dynamical impact of blocking the M-current on slow oscillations, both in cortical slices and in a cortical network computational model. Blocking M-current resulted in an elongation of Up states (by four times) and in a significant firing rate increase, reflecting an increased network excitability, albeit no epileptiform discharges occurred. These effects were replicated in a biophysical cortical model, where a parametric reduction of the M-current resulted in a progressive elongation of Up states and firing rate. All neurons, and not only those modeled with M-current, increased their firing rates due to network recurrency. Further increases in excitability induced even longer Up states, approaching the microarousals described in the transition towards wakefulness. Our results bridge an ionic current with network modulation, providing a mechanistic insight into network dynamics of awakening.

## Author summary

During slow wave sleep we are unconscious and disconnected from the outside world, while when we wake we are conscious of ourselves and the world around us. These two different brain states, slow wave sleep and wakefulness, are rather different from a network dynamics perspective: highly synchronous and desynchronized, respectively. Furthermore, spatiotemporal complexity of activity patterns is low for slow wave sleep and high for wakefulness. What are the mechanisms driving the transition between these very different functional brain states? In the current study we investigated one mechanism that is critical for the sleep to wake transition to occur in the cerebral cortex network: the blockade of M-current by cholinergic action, and we did this both in experiments (*in vitro*) and in a computational model (*in silico*). We observed that the slow oscillations that dominate the synchronous activity are largely transformed by M-current blockade, the periods of activity or Up states becoming longer, and eventually generating microarousals that lead into wakefulness. In this study we bridge ionic channels, network dynamics, and brain states to understand how microscopic mechanisms determine macroscopic brain states and information processing capabilities.

## Introduction

Slow oscillations or slow wave dynamics are a highly synchronized activity pattern that dominate the cerebral cortex dynamics in slow wave sleep and deep anesthesia [1–3]. Moreover, slow oscillations have been observed to arise in certain pathological conditions, such as disorders of consciousness [4], while the presence of delta waves during wakefulness has long been regarded as a diagnostic biomarker for lesions such as stroke [5]. Slow waves have more recently been reported in the areas around stroke lesions and connected areas [6], and even around the focal thermocoagulation lesions in the treatment of epilepsy [7]. Interestingly, slow oscillations can be considered a circuit attractor for cortical dynamics [3] as well as the default activity pattern of the cerebral cortex network [8]: when cortical circuits get structurally or functionally disconnected, slow oscillations emerge, an example being the cortical slices maintained *in vitro* [9], in cortical slabs [10], or in perilesional tissue [6]. The ubiquity of this slow rhythm makes it relevant for understanding the underlying mechanisms and modulatory mechanisms in both physiological and pathological conditions.

Slow wave activity is a state of cortical bistability, characterized by the spontaneous and almost periodic emergence of Up (active; neuronal depolarization and firing) and Down (silent; neuronal hyperpolarization) states, in which network interactions are disrupted [11], complexity is low [12,13], synchrony is high [14], and information processing is therefore impaired. In order for cortical circuits to be in an asynchronous state that can process information [15] the system should leave the slow wave attractor [16,17] and recover spatiotemporal dynamics and properties that support wakefulness [18]. The physiological process for the transition from synchronous (slow waves) to asynchronous states (wakefulness)requires the action of various neurotransmitters, amongst which acetylcholine (ACh) has a prominent role [19–21]. An important effector of ACh is the muscarinic blockade of muscarinic-sensitive potassium current (M-current) [22], a non-inactivating potassium current that controls membrane excitability. The blockade of M-current lowers the threshold for firing and increases the cellular input resistance [23,24], but its effect on network dynamics is unknown.

Here, we sought to determine the impact of M-current on cortical dynamics, specifically during spontaneous Up and Down states, and both experimentally and in a computational model. We studied the effects of the M-current blocker XE991 (100 μM) on the slow oscillatory regime. In a computational model, we reproduced the experimental observations and explored more areas of the parameter space, along with a parametric variation of M-current (enhancing and reducing) and resulting impact on population emergent patterns. Overall, our findings highlight the crucial role of M-current in regulating cortical network activity and Up states’ duration and suggest that its blockade, mimicking the effects of acetylcholine, may play a key role in the neural mechanisms in the process of awakening.

## Results

We recorded local field potentials (LFPs) from ferret cerebral cortex slices (*n* = 10) that generate spontaneous slow oscillations, a network emergent pattern consisting of interspersed Up (active) and Down (almost silent) states. To investigate the role of M-current on this network activity, we blocked neuronal Kv7 channels (also known as KCNQ channels), which are the molecular correlates of the M-current. To this end, we bath-applied XE991 dihydrochloride, a specific KCNQ2/3 channel blocker of M-current [25,26]. We aimed to completely block the current [27], using a concentration of XE991 of 100 μM.

**Fig 1:**
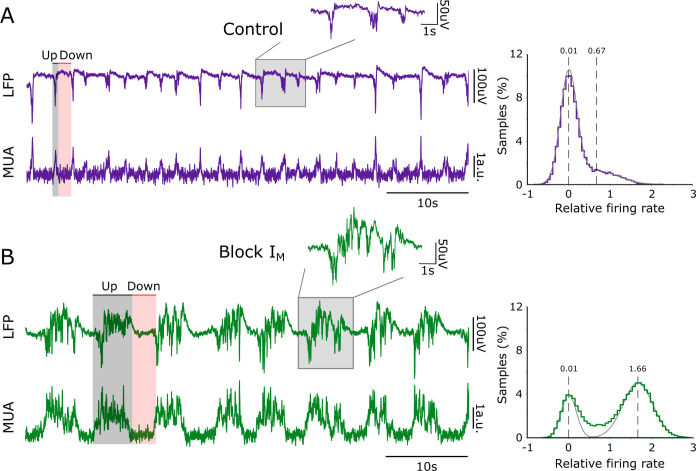
Effect of M-current blocker on spontaneous slow oscillations. (A) Raw local field potential (LFP) and relative firing rate (MUA; see Methods) illustrating network activity in neocortical slices during control slow oscillation. Up (gray shadow) and Down (red shadow) states were detected from the relative firing rate. Right: histogram of the relative firing rate. The gray dotted line represents the Gaussian mean of the lower and upper distribution tails, respectively, aiming at representing the activity distribution of Down and Up states. (B) Spontaneous network activity after blockade of M-current (I_M_).

**Fig 2:**
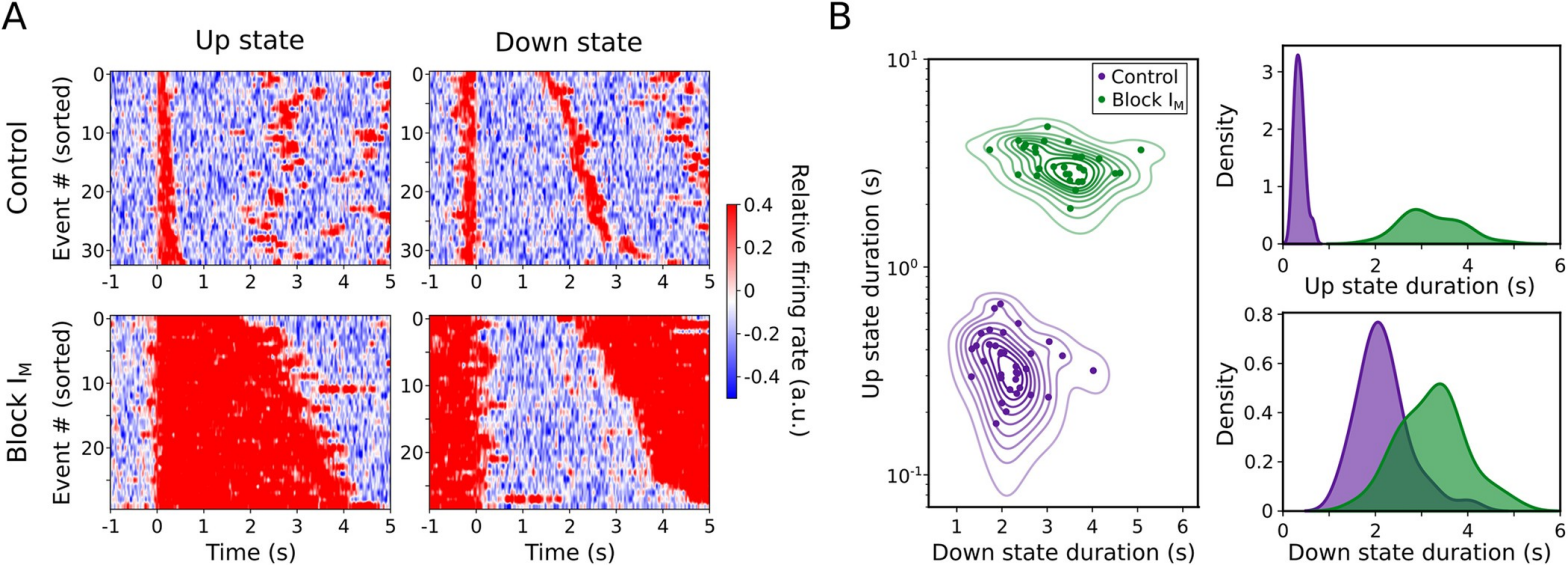
Modulation of Up and Down states through the blockade of M-current in an illustrative single case. (A) Raster plots of relative firing rate (MUA) of all Up and Down states detected in control (top row) and blockade of M-current (I_M_; bottom row). (B) Scatter plot of Up and Down durations. Irregular ellipses stand for the bivariate (2D) kernel density estimated (KDE; see Methods for details).

**Fig 3:**
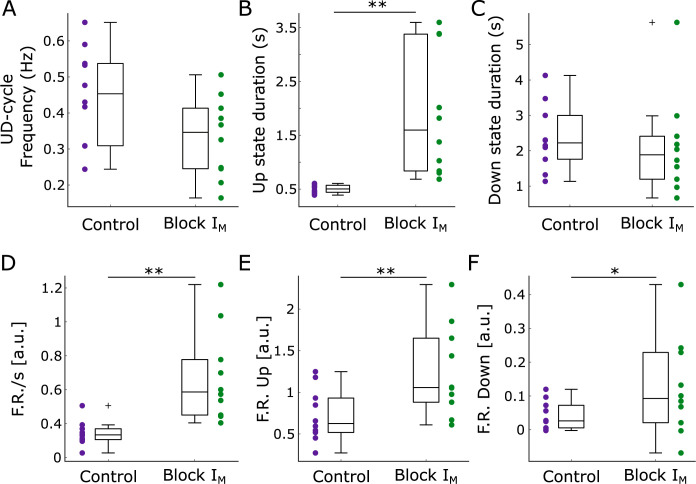
Relative changes of Up and Down state properties during the M-current blockage. (A) Frequency of the Up and Down cycle (UD-cycle). (B) Up state duration. (C) Down state duration. (D) Relative firing rate per second. (E) Relative firing rate during Up states. (F) Relative firing rate during Down states. Relative firing rate is defined as the mean MUA across time (see Methods). * *p*<0.05; ** *p*<0.01 (two-sided Wilcoxon signed-rank test).

The baseline frequency of the slow oscillations in our experimental sample was 0.44 Hz (0.44 ± 0.13 Hz, mean ± SD), with an Up and Down state duration of 0.50 ± 0.07 s and 2.36 ± 0.93 s, respectively (Fig 1A). Local field potential (LFP) and the corresponding multiunit activity (MUA) are displayed (see Methods; calculated as in [28]). XE991 was applied to the bath at a concentration of 100 μM, resulting in prominent changes in the spontaneous emergent activity from the cortical network (Table 1): remarkably, elongated Up states are evident in the example displayed in Fig 1. The main changes resulting from blocking M-current were an increase in the population firing rate (computed as the normalized MUA spectrum at high frequencies, 0.2–1.5 kHz, as in [25]; see Methods for details) during Up and Down states and a prominent elongation of the Up states (Control: 0.50 ± 0.07 s, Block I_M_: 1.89 ± 1.16 s). Fig 2 represents the elongation of Up states in raster plots for a single slice (Fig 2A), and in the duration’s histogram (Fig 2B). A slight elongation of Down states was expressed in this case, although at the population level there was no significant change in the duration of Down states as a result of M-current blockade (Fig 3 and Table 1). By plotting the Up state duration versus the Down state duration, two well-separated clusters are observed, highlighting the effect on the Up states after the blockade of the M-current (Fig 2B).

The increase in the population firing rate (Fig 3D–3F), confirms the role of M-current in the control of network excitability. A significant increment in the network firing rate both during Up and Down states was observed after the blockade of M-current. Much as M-current has a high impact on the network excitability, epileptiform discharges were never observed.

**Table 1:** Relative changes of Up and Down state properties during the blockade of M-current. *P*-value of a two-sided Wilcoxon signed rank test (*p<0.05, **p<0.01).

Parameter	Control (mean±sd)	XE991 (mean±sd)	*p*-value
Frequency (Hz)	0.44±0.13	0.33±0.13	0.06
Up state duration (s)	0.50±0.07	1.89±1.16	0.002**
Down state duration (s)	2.36±0.93	2.13±1.41	0.37
Up state relative firing rate	0.72±0.31	1.24±0.54	0.009**
Down state relative firing rate	0.04±0.04	0.12±0.14	0.04*
Relative firing rate per second	0.18±0.09	0.52±0.38	0.002**

### Network model

**Fig 4:**
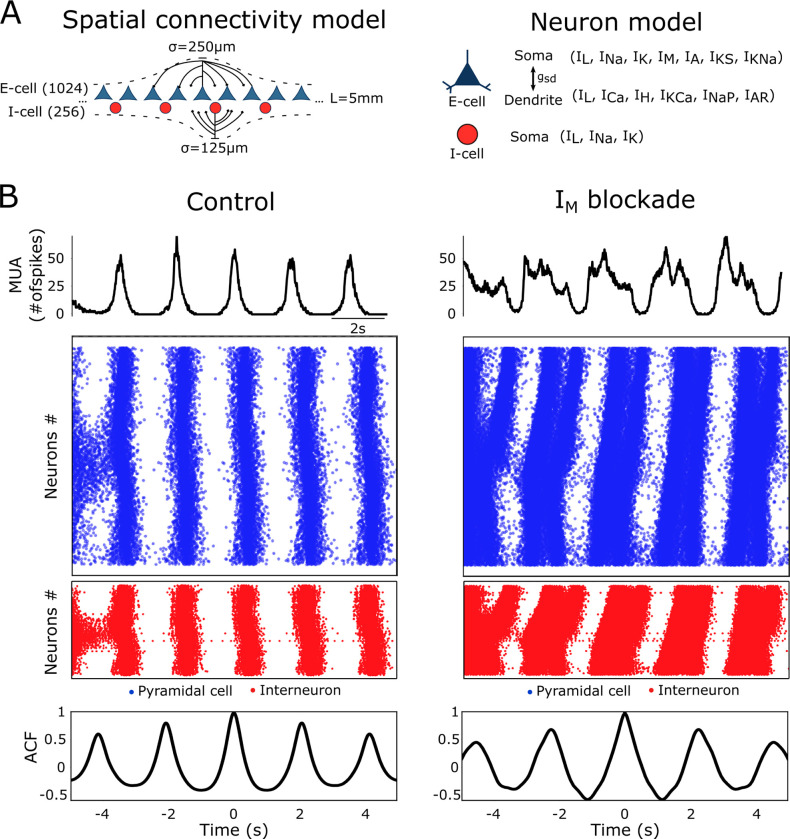
M-current on the cortical network model. (A) Schematic representation of the network spatial connectivity and the neuron model. Our network is assumed to be 5 mm long and is composed of pyramidal cells (E-cells; blue) and interneurons (I-cells; red). The probability of connection between neurons are given by a Gaussian probability distribution with distance decay, centered at each neuron, and with a prescribed standard deviation σ (see Methods for details). The pyramidal neurons are modeled as a two-compartment model (soma and dendrite) while the interneurons are modeled as single compartment. (B) Left column: slow oscillations (SO) activity in the form of Up and Down states in a control network visualized as the sum of network activity (top) and raster plot (middle). The autocorrelation function (ACF) of the firing rate is shown at the bottom. Right column: effect of M-current (I_M_) blockade (80%) on slow oscillatory activity.

To carry out a detailed, mechanistic, and quantitative exploration of the role of M-current (I_M_) in the cortical network, we implemented a biophysically detailed computational model of the cortical network [29]. The model consists of pyramidal and inhibitory conductance-based neurons equidistantly distributed on a line and interconnected through biologically plausible synaptic dynamics. In the network, neurons are sparsely connected with a probability that decays with the distance between them (Fig 4A). This, together with some randomly distributed intrinsic parameters, are the only source of noise in the model; that is, neurons do not receive any other external input. This implementation allows us to have a heterogeneous network with cell-to-cell variability, as experimentally observed (for a review see [30]). Additionally, our model accounts for the M-current as described in [31] and the H-current as described in [32].

**Fig 5:**
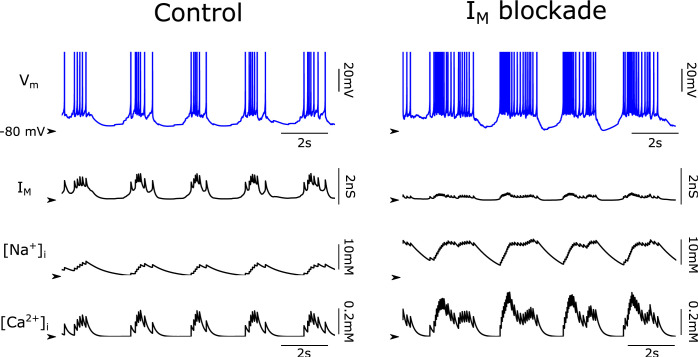
Impact of M-current at the neuronal and ionic level. Representative membrane potential of the pyramidal cells during control slow oscillations (left column) and under blockade (80%) of M-current (I_M_; right column). The dynamics of M-current are shown together with the Na^+^ and Ca^2+^ intracellular concentration. The arrows point to 0nS for I_M_, 10mM to [Na^+^]_i_ and 0mM to [Ca^2+^]_i_.

**Fig 6:**
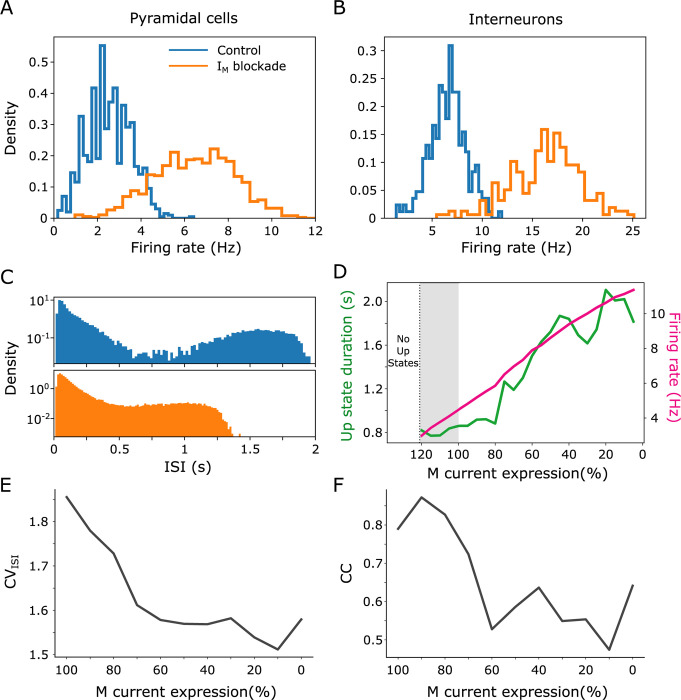
Network features under M-current blockade in the cortical network model. (A) and (B), neuronal firing rate distribution during control (slow oscillations, SO; blue) and blockade (80%; orange) of M-current (I_M_) for pyramidal cells and interneurons, respectively. (C) Interspike interval distribution (ISI) for pyramidal cells during SO (blue) and I_M_ blockade (orange). (D) Up state duration (green) and neuronal firing rate (pink) as a function of I_M_ expression, respectively. Gray shadow marks the region where we increased the I_M_ expression from control. Above 20% of control expression the network is unable to display spontaneous activity in the form of Up states. (E) Coefficient of variation (CV_ISI_) of the interspike interval averaged over all neurons. (F) Averaged pairwise cross-correlation (CC) across neurons pairs.

Our model was able to reproduce the Up (active state) and Down (silent state) dynamics observed during slow oscillations as well as the activity under blockade of M-current (Figs 4 and 5). To explore the M-current effect on the model we parametrically decreased its maximal channel conductance (*g*_*M*_) from 100% to 10% in the pyramidal neurons (see Methods for details). The neuronal firing is represented in raster plots for both pyramidal cells (blue) and inhibitory interneurons (red). Both display an elongation of the Up states under M-current block. Even though interneurons do not express M-current, they are recurrently connected with pyramidal neurons. The periodic alternation between Up and Down states during control slow oscillations (Fig 4) became slightly more irregular after the blockade of M-current (compare autocorrelation function, ACF, in Fig 4, left and right columns; and it has been further quantified in Fig 6E and 6F).

The neuronal membrane potential during slow oscillations displayed the typical dynamics of pyramidal cells during this regime (Fig 5) [1,9]. There was an increase in the M-current following each action potential, as well as an increase in the intracellular concentration of sodium and calcium (Fig 5). These intracellular ions then activated the afterhyperpolarization (AHP) currents sodium-dependent potassium current (I_KNa_) and calcium-dependent potassium current (I_KCa_) (Fig 5; [29,33]). When network excitability decreased due to the negative feedback generated by the AHP currents, the periods of high activity (Up states) could no longer be sustained, and the network fell into a silent (Down) state. During the Down states, the AHP currents started to decay and eventually the network, due to its recurrent excitation, was able to trigger a new Up state (Fig 5). When the M-current expression was reduced in the network, the neurons also displayed periods of activation and silence (Down state); however, the neuronal discharges were longer than those observed in the control situation. Due to the prolonged time of neuronal discharge, as well as to the higher firing rates, the concentration of intracellular sodium and calcium was higher than those observed during control slow oscillations.

Seeking to better understand the effects of M-current on spontaneous slow oscillations we carried out a parametric variation in the expression of M-current. As shown in Fig 6, the neuronal firing rate increased when the M-current was decreased, not only for pyramidal neurons (Control: 3.02 ± 0.99 Hz, 80% M blockage: 6.81 ± 1.94 Hz), but also for inhibitory neurons (Control: 7.04 ± 1.74 Hz, 80% M blockage: 16.29 ± 3.39 Hz), that did not contain the M-current (Fig 6A and 6B). The interspike interval (ISI) distribution for the control condition of SO showed a lack of intermediate time values, which are typical of slow oscillations and reflect the periods of silence (Down states). Conversely, when M-current was decreased, the periods of silence were not reflected in the ISI distribution (Fig 6C), and a more irregular activity was observed, as quantified by the ISI’s coefficient of variation (CV_ISI_) and by the pairwise cross-correlation (CC; Fig 6E and 6F). By evaluating the Up state duration and neuronal firing rate as a function of the M-current expression we observed a linear relationship such that the lower the expression, the higher the duration of Up states and the firing rate (Fig 6D). Furthermore, we also tested the effects of enhancement of M-current in the model. We were able to increase its expression by 20%, and the linear relationship between Up state duration and neuronal firing rate was maintained. For an increment greater than 20% the network was unable to generate spontaneous activity (Fig 6D).

**Fig 7:**
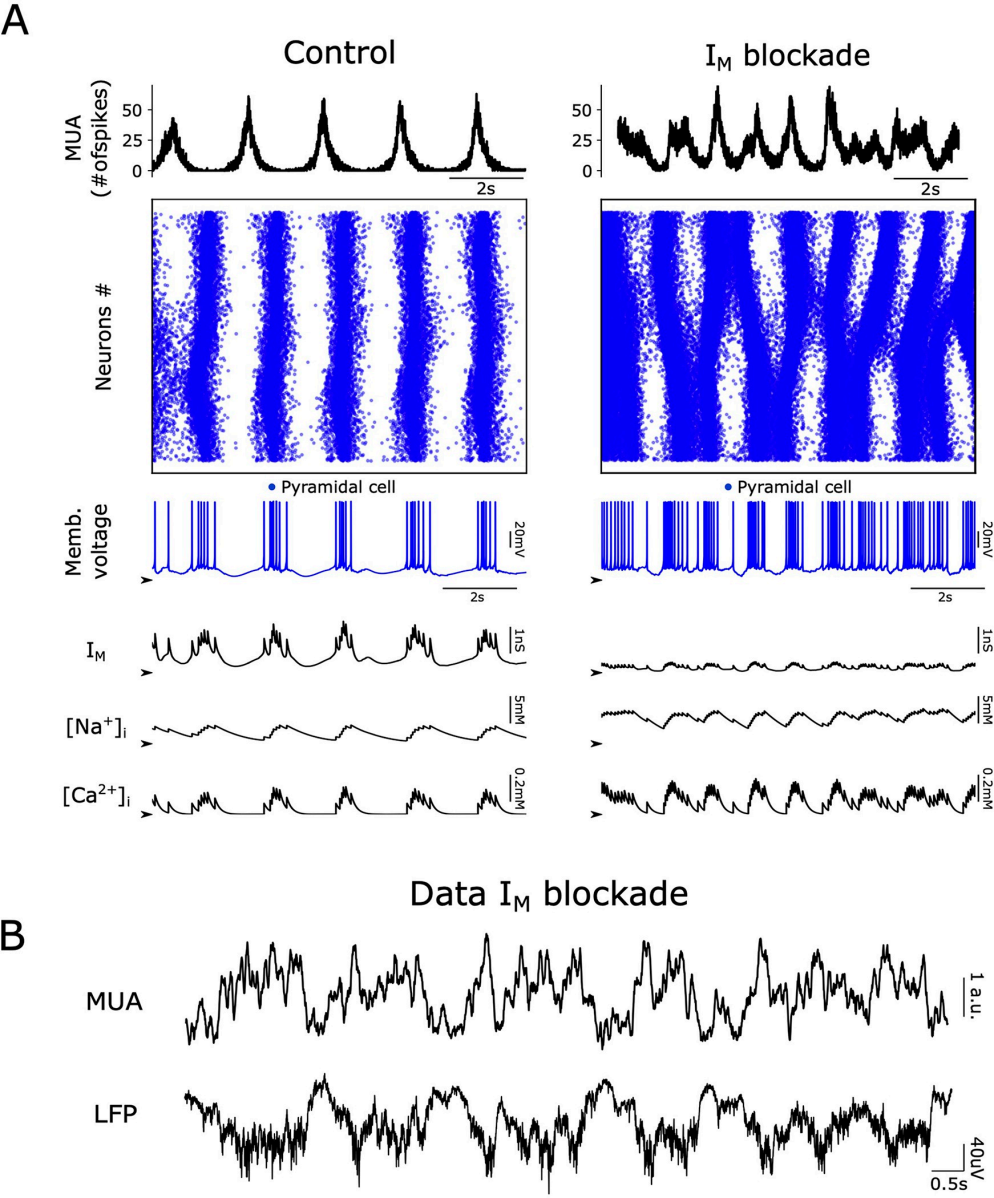
Impact of M-current on a more global excitable network. (A) Representation of the network model in a more excitable state, during control activity (left column) and during the M-current (I_M_) blockade (right column). Such states can be achieved by decreasing the leak current reversal potential (here reduced to −60.85 mV; see S1 Fig), for example, and its dynamics are more irregular (compare with Fig 4B). (B) In the experimental data, more irregular dynamics may also be observed after blockade of M-current.

In an active brain, neuronal excitability is not constant, but it varies following extracellular ionic concentrations and presence of neurotransmitters. For this reason, we investigated the impact of blocking M-current in a situation of higher network excitability in the model, that was achieved by reducing leak current varying the I_L_ reversal potential. Following the blockade of M-current, the network did not show clear Up and Down states in a state of higher excitability, but longer periods of persistent activity (Fig 7). In such networks, single neurons showed a pattern of activity reminiscent of the microarousals [34] (Fig 7; see also recordings in [35]), and irregular propagation waves were observed through the network, resembling the patterns generated by ACh modulation [20, 21]. Similar results were obtained when the excitability was increased by depolarizing excitatory neurons by current injection (S1 and S2 Figs). Our experimental data also showed occasional periods of spontaneous activity where more irregular dynamics were observed and in which persistent activity dominated over the regular occurrence of Down states (Fig 7B), reminiscent of similar observations reported *in vivo* in the transition towards wakefulness [15, fig 5A in 47].

## Discussion

In the current work we investigated the effect of reducing M-current over a physiological network activity of the cerebral cortex: slow oscillations. Slow oscillations not only occur in slow wave sleep and in deep anesthesia (for a review see [2,3]) but also in isolated cortical tissue, such as isolated cortical gyri, cortical slabs [10], cortical slices [9], or tissue disconnected by injury [6]. Such pervasive activity acts as the default activity of the cortical circuitry [8], emerging as a result of the integration of intrinsic properties of neurons such as ionic channels, cellular and synaptic properties and connectivity. We carried out a study in cortical slices expressing spontaneous slow oscillatory activity *in vitro*, allowing us to isolate the effect of M-current on the cortical network, in the absence of the influence of other connected brain areas.

M-current is a time- and voltage-dependent K^+^ current, which is non-inactivating and non-rectifying, and exerts a clamping effect of the membrane potential [22]. The molecular basis of this current was identified by Wang et al. [25], corresponding to the KCNQ2 and KCNQ3 potassium channels. Because the M-current is a powerful stabilizer of the membrane potential, controlling subthreshold activity and synaptic responses, abnormal function of neuronal KCNQ channels have been associated with diseases related to hyperexcitability: a loss of just 25% of KCNQ2 or KCNQ3 channels are the cause of benign familial neonatal seizures [36,37]. The control of neuronal excitability by M-current has also been exploited as a therapeutic strategy, enhancing this current for the treatment of epilepsy [38] or to reduce stroke-related brain injury [39].

The effects of blocking M-current were originally described in bullfrog sympathetic neurons [40]. Specific blockade was caused by muscarine and by other agents, resulting in an inward rectification causing depolarization, increased input resistance, reduced outward rectification, and increased excitability. Such an increase in excitability has also been observed in our study in which we used XE991 dihydrochloride (100 μM) as a specific M-current blocker [27] to explore the impact on slow oscillations. We found that M-current has an important role in the mechanisms controlling the Up state, specifically modulating its persistence and termination. Blocking the M-current resulted in a prominent elongation (ca. four times) of the periods of activation (Up states) of the cortical network, while the Down states or silent periods hardly varied in duration. The population firing rate was also significantly increased during both Up and Down states, also reflecting the hyperexcitability of the network. It should be noted that this excitability includes depolarization of the membrane potential, but also an increase in input resistance, such that the synaptic inputs evoke larger responses [40,41]. Given that Up states are driven by bursts of synaptic inputs from neighboring neurons [1], global activity is enhanced. The effect on Up states is very prominent, prolonging them to the point that they are similar to the so-called microarousals (Fig 7) that occur during slow wave sleep as a result of activation of the arousal systems [34] and also appear along with slow oscillations in the transition from slow wave dominated, deep anesthesia, towards light anesthesia [16].

Interestingly, the blockade of M-current in the isolated neocortical network does not result in epilepsy, as occurs in the case of neonates with deficits in the current [36] or in transgenic mice with M-current suppression [42]. We suggest that the recurrent connections with more epileptogenic areas in these circumstances, such as the hippocampus [42,43], can drive the neocortex *in vivo* into epileptic discharges. However, such epileptic discharges do not seem to be originated through this mechanism in the cortical network, not even in highly excitable cortical areas like the entorhinal cortex [44].

In order to do a systematic exploration of the role of M-current on the network, by varying its expression parametrically we reproduced a conductance-based computational model of the cortical network that expresses slow oscillations [29]. In it, the M-current was newly integrated in the excitatory neurons following the model in [31] and locating it in the somatic compartment [45]. In this new implementation of the model, we were able to reproduce the physiological Up and Down dynamics of the slow oscillations as well as the experimental effects of blocking the M-current. As observed in the experiments, blocking the M-current in the model resulted in an increase in firing rate. Interestingly, even when only the excitatory neurons expressed M-current, the increase in firing rate also occurred in inhibitory neurons, by virtue of recurrent connectivity. By parametrically blocking the M-current (from 0% to 90%), we found a correlation between the elongation of the Up states and the increase in firing rate, such that the lower the expression, the higher the duration of Up states and the firing rate (Fig 6).

The role of M-current blockade in the modulation of firing and synchronization of the network is highly relevant if we consider that this current owes its name to “muscarine”: in 1980, Brown and Adams discovered a novel voltage-sensitive K^+^ current that was suppressed by muscarine, and that they called M-current [22]. Indeed, since McCormick and Prince [41], a large part of the studies of M-current in the cerebral cortex have been carried out in the context of studying the impact of cholinergic innervation. Acetylcholine is one of the main neurotransmitters inducing the transition from slow wave sleep to wakefulness. In particular, cholinergic action in the cerebral cortex takes place largely through muscarinic rather than nicotinic receptors, and as shown here, an important actor (but not the only one) is blockade of the M-current. As generated in our computational model at the individual neuron level (Fig 5), just the reduction of this current induces quite a radical change in network dynamics, which goes from regular and synchronous slow oscillations to more prolonged and slightly more irregular firing periods. However, this is not yet the asynchronous, persistent activated state associated with wakefulness [46], because there are other potassium channels (sodium- and calcium-dependent potassium currents) that remain open, repolarizing the membrane potential towards Down states (Fig 5). The similarity of this activity pattern with those described in the transition periods towards wakefulness that are achieved by light (versus deep) levels of anesthesia, when microarousals occur, is remarkable (fig 5A in [47]).

In conclusion, our experimental results together with our computational model, support the conclusion that M-current plays a highly relevant role in cortical network dynamics. This effect is even more relevant if we consider that probably only a fraction of neurons express this current, but its effects reverberate through recurrent connectivity. The blockade of M-current induces a significant increased excitability, with longer and synchronous periods of activity or Up states and higher firing rates. Since cholinergic action in the cerebral cortex is critical to induce the transition from slow wave sleep to wakefulness, our observations suggest a relevant role for M-current blockade by muscarinic action into this transition. On the contrary, the physiological activation of the M-current plays an important role in maintaining a hyperpolarized neuronal membrane potential and facilitating, in the absence of cholinergic inputs, the expression of slow waves in the cortical network.

## Materials and methods

### Ethics statement

Ferrets were treated in accordance with the European Union guidelines on the protection of vertebrates used for experimentation (Directive 2010/63/EU of the European Parliament and of the council of 22 September 2010). All experiments were approved by the ethics committee of the University of Barcelona. Several elements of the methods described here are taken from and described in our previous work [17].

### Slice preparation

Ferrets (4–10-months-old; either sex) were deeply anesthetized with isoflurane and sodium pentobarbital (40 mg/kg) before decapitation. The brain was quickly removed and placed in an ice-cold sucrose solution containing (in mM): 213 sucrose, 2.5 KCl, 1 NaH_2_PO_4_, 26 NaHCO_3_, 1 CaCl_2_, 3 MgSO_4_ and 10 glucose and acute coronal slices (400-μm-thick) of the occipital cortex containing visual cortical areas 17, 18, and 19 from both hemispheres were obtained.

Slices were placed in an interface-style recording chamber (Fine Science Tools, Foster City, CA) and superfused with an equal mixture of the above-mentioned sucrose solution and artificial cerebrospinal fluid (ACSF) as in [9]. The ACSF contained (in mM): 126 NaCl, 2.5 KCl, 1 NaH_2_PO_4_, 26 NaHCO_3_, 2 CaCl_2_, 2 MgSO_4_ and 10 glucose. Next, slices were bathed with ACSF for 1–2 h to allow recovery. For slow oscillatory activity to spontaneously emerge, slices were superfused for at least 30 min before experiments with ACSF containing (in mM): 126 NaCl, 4 KCl, 1 NaH_2_PO_4_, 26 NaHCO_3_, 1 CaCl_2_, 1 MgSO_4_ and 10 glucose. All solutions were saturated with carbogen (95% O_2_/5% CO_2_) to a final pH of 7.4 at 34°C.

### Electrophysiological recordings

We recorded the extracellular local field potential (LFP) using a 16-channel SU-8-based flexible microarray [48,49]. Signals were amplified by 100 using a PGA16 Multichannel System (Multichannel Systems MCS GmbH-Harvard Bioscience Inc, Reutlingen, Germany). LFPs were digitized with a Power 1401 or 1401 mkII CED interface (Cambridge Electronic Design, Cambridge, UK) at a sampling rate of 5 or 10 kHz and acquired with Spike2 software (Cambridge Electronic Design, Cambridge, UK).

### Pharmacological agents

XE991 dihydrochloride (100 μM) was used as a specific M-current blocker and was obtained from Tocris Bioscience (UK).

### Computational modeling

For a quantitative investigation and analysis of the effects of M-current in the modulation of slow oscillations, we simulated the model of isolated cortical network proposed by Compte et al. [29], with the addition of the M-current. The model consists of 1024 pyramidal neurons and 256 inhibitory neurons interconnected through biologically plausible synaptic dynamics. The neurons are equidistantly distributed on a line and sparsely connected to each other, as in the control network described in [29]. Each neuron makes a 20±5 (SD) connection to its postsynaptic targets (autapses are not allowed). The network is assumed to be 5 mm long and the probability of connections between neuron pairs is determined by a Gaussian distribution centered at zero with a prescribed standard deviation. For pyramidal and inhibitory neurons, the standard deviation was set at 250 μm and 125 μm, respectively. For more details see [29].

Pyramidal neurons have a somatic and a dendritic compartment. The dynamical equations are:

$$C_{m}A_{s}\frac{dV_{s}}{dt}=-A_{s}(I_{L}+I_{Na}+I_{K}+I_{A}+I_{KS}+I_{KNa}+I_{M})-I_{syn,I}-g_{sd}(V_{s}-V_{d}),$$

and

$$C_{m}A_{d}\frac{dV_{d}}{dt}=-A_{d}(I_{L}+I_{Ca}+I_{H}+I_{NaP}+I_{AR}+I_{KCa})-I_{syn,E}-g_{sd}(V_{d}-V_{s}).$$

*V*_*S*_(*V*_*d*_) and *A*_*S*_ = 0.015 mm^2^ (*A*_*d*_ = 0.035 mm^2^) represent the soma (dendrite) membrane voltage and membrane area, respectively. *C*_*m*_ = 1μF/cm^2^ is the specific membrane capacitance and *g*_*sd*_ = 1.75±0.1μS is the electrical coupling conductance between soma and dendrite. *I*_*syn*,*E*_ (*I*_*syn*,*I*_) are the excitatory (inhibitory) synaptic currents. Following Compte et al. [29], in our simulations, all excitatory synapses target the dendritic compartment and all inhibitory synapses are localized on the somatic compartment of postsynaptic pyramidal neurons.

The somatic compartment includes the following channels and respective maximal conductance (g): leakage current (*I*_*L*_, *g*_*L*_ = 0.0667±0.0067 mS/cm^2^), sodium current (*I*_*Na*_, *g*_*Na*_ = 50 mS/cm^2^), potassium current (*I*_*K*_, *g*_*K*_ = 10.5 mS/cm^2^), A-type K^+^ current (*I*_*A*_, *g*_*A*_ = 0.9 mS/cm^2^), non-inactivating slow K^+^ current (*I*_*KS*_, *g*_*KS*_ = 0.403 mS/cm^2^), Na^+^-dependent K^+^ current (*I*_*KNa*_, *g*_*KNa*_ = 0.744 mS/cm^2^), and the non-inactivating and non-rectifying K^+^ current (*I*_*M*_, *g*_*M*_ = 0.083 mS/cm^2^). The dendrite includes leakage current (*I*_*L*_, *g*_*L*_ = 0.0667±0.0067 mS/cm^2^), high-threshold Ca^2+^ (*I*_*Ca*_, *g*_*Ca*_ = 0.43 mS/cm^2^), non-inactivating hyperpolarization-activated current (*I*_*H*_, *g*_*H*_ = 0.0115 mS/cm^2^), persistent Na^+^ channel (*I*_*NaP*_, *g*_*NaP*_ = 0.0686 mS/cm^2^), anomalous rectifier K^+^ channel (*I*_*AR*_, *g*_*AR*_ = 0.0257 mS/cm^2^), and the Ca^2+^-dependent K^+^ current (*I*_*KCa*_, *g*_*KCa*_ = 0.57 mS/cm^2^). The inhibitory neurons, consisting of only a single compartment, are simply modeled as:

$$C_{m}A_{i}\frac{dV_{s}}{dt}=-A_{i}(I_{L}+I_{Na}+I_{K})-I_{syn},$$

where *A*_*i*_ = 0.02mm^2^ is the total membrane area and *I*_*syn*_ is the synaptic current from both the inhibitory and excitatory neurons. The maximal conductances are: *g*_*L*_ = 0.1025±0.0025 mS/cm^2^, *g*_*Na*_ = 35 mS/cm^2^, and *g*_*K*_ = 9 mS/cm^2^. All the details of the implementation of these currents are described in [29], except for *I*_*H*_ and *I*_*M*_, which are described below. For the M-current we implemented the model described by McCormick et al. [31]: *I*_*M*_ = *g*_*M*_*m*(*V*−*V*_*K*_). The activation variable is controlled by $m_{\infty }=\frac{1}{1+exp[-0.1(V+35)]}$ and $\tau_{m}=\frac{1000}{3.3exp[(V+35)/20]+exp[-(V+35)/20]}$. For the H-current we implemented the model described by Hill et al. [32]: *I*_*H*_ = *g*_*H*_*m*(*V*+45). The activation variable is controlled by $m_{\infty }=\frac{1}{1+exp[(V+75)/5.5]}$ and $\tau_{m}=\frac{1}{exp[-14.59-0.086V]+exp[-1.87+0.0701V]}$. To simulate the experimental effects of M-current blockage, we progressively reduced the *I*_*M*_ maximal conductance (*g*_*M*_) from 100% (control condition) to 10% (which will be referred to as simple concentration). The synaptic currents were modeled as described in [29] with the following adjustments of AMPA, NMDA and GABA-A maximal conductance: $g_{EE}^{AMPA}=3\;nS,\;g_{EI}^{AMPA}=2.25\;nS,\;g_{EE}^{NMDA}=0.9\;nS,\;g_{EI}^{NMDA}=0.5\;nS,\;g_{II}^{GABA}=0.66\;nS$, and $g_{IE}^{GABA}=4.15\;nS$.

### Network dynamics analysis

For every condition tested, we analyzed 200 s of spontaneous activity. We first estimated multi-unit activity (MUA) from LFP recordings and detected Up and Down states as previously described [13,50,51]. Briefly, the MUA signal was calculated as the average power of the normalized spectra at high-frequency band (200–1500 Hz), since power variations in the Fourier components at high frequencies of LFP provide a reliable estimate of the population firing rate [52]. The power spectra of the extracellular recording were computed every 5 ms and their average during Down states was chosen as baseline for normalization, resulting in the “relative firing rate”. The MUA signal was logarithmically scaled to balance large fluctuations of nearby spikes. We detected Up and Down states setting duration and amplitude thresholds in the log(MUA) signal. In this way, we could compute different parameters that characterize SO, such as oscillation frequency or Up and Down state durations. For each Up and Down cycle, the duration for both the Up and Down states were computed. Once extracted, they were scattered one against the other to construct the 2D space of points coloring by condition. Kernel density estimation (KDE) was used to obtain and construct univariate (1D histogram estimate) and bivariate (2D histogram estimate) plots of the Up and Down state durations. For the simulated data, the Up states were detected thresholding the Gaussian smoothed network activity (defined as the sum of spikes within a 4 ms bin). Network regularity was computed as the coefficient of variation of the interspike interval (CV = 1 stands for Poisson-like dynamics) and by the pairwise cross-correlation (CC = 1 stands for a highly synchronized network, while CC = 0 for an asynchronous network spiking activity) as in [53,54].

## Supporting information

S1 Fig Effects of M-current concentration (left), leak reversal potential (middle) and depolarizing current (right) on the resting membrane potential of an excitatory neuron.
(JPEG)

S2 Fig Impact of M-current blockade in a more excitable network. Higher excitability is achieved by a depolarizing current in all excitatory neurons (0.02 nA). Left column: control activity. Right column: M-current blockade (80%).
(JPEG)
