# Supplementary figures and images for: M-current modulation of cortical slow oscillations: Network dynamics and computational modeling
Source: PLoS Comput Biol. 2023 Jul 5;19(7):e1011246. doi: 10.1371/journal.pcbi.1011246 (PMC10351697; doi:10.1371/journal.pcbi.1011246)

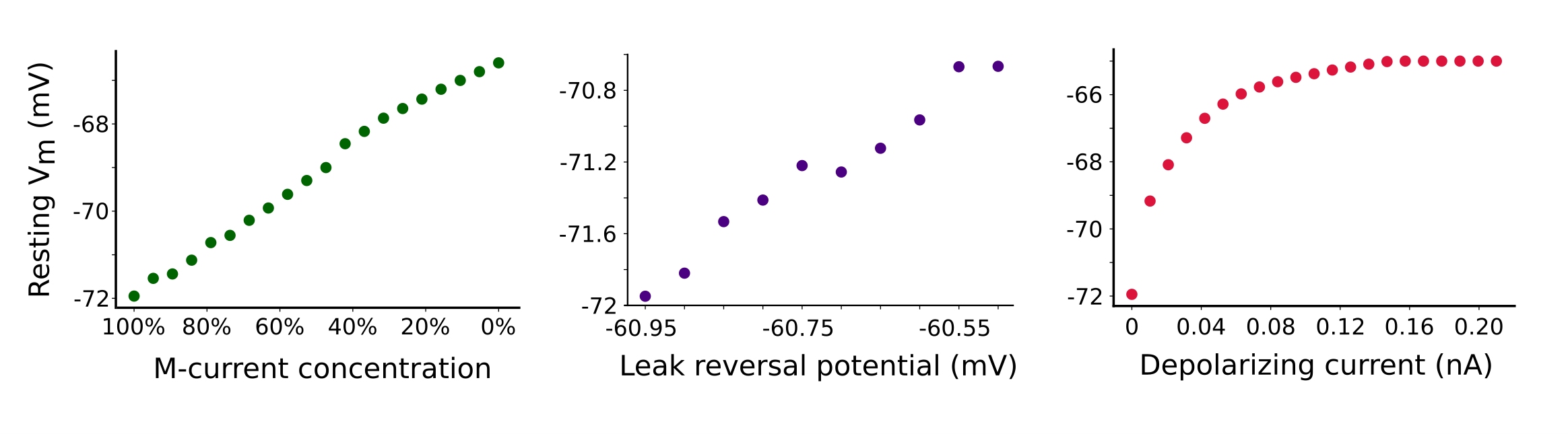

Supplement: S1 Fig — Effects of M-current concentration (left), leak reversal potential (middle) and depolarizing current (right) on the resting membrane potential of an excitatory neuron. (JPEG) [file pcbi.1011246.s001.jpeg]

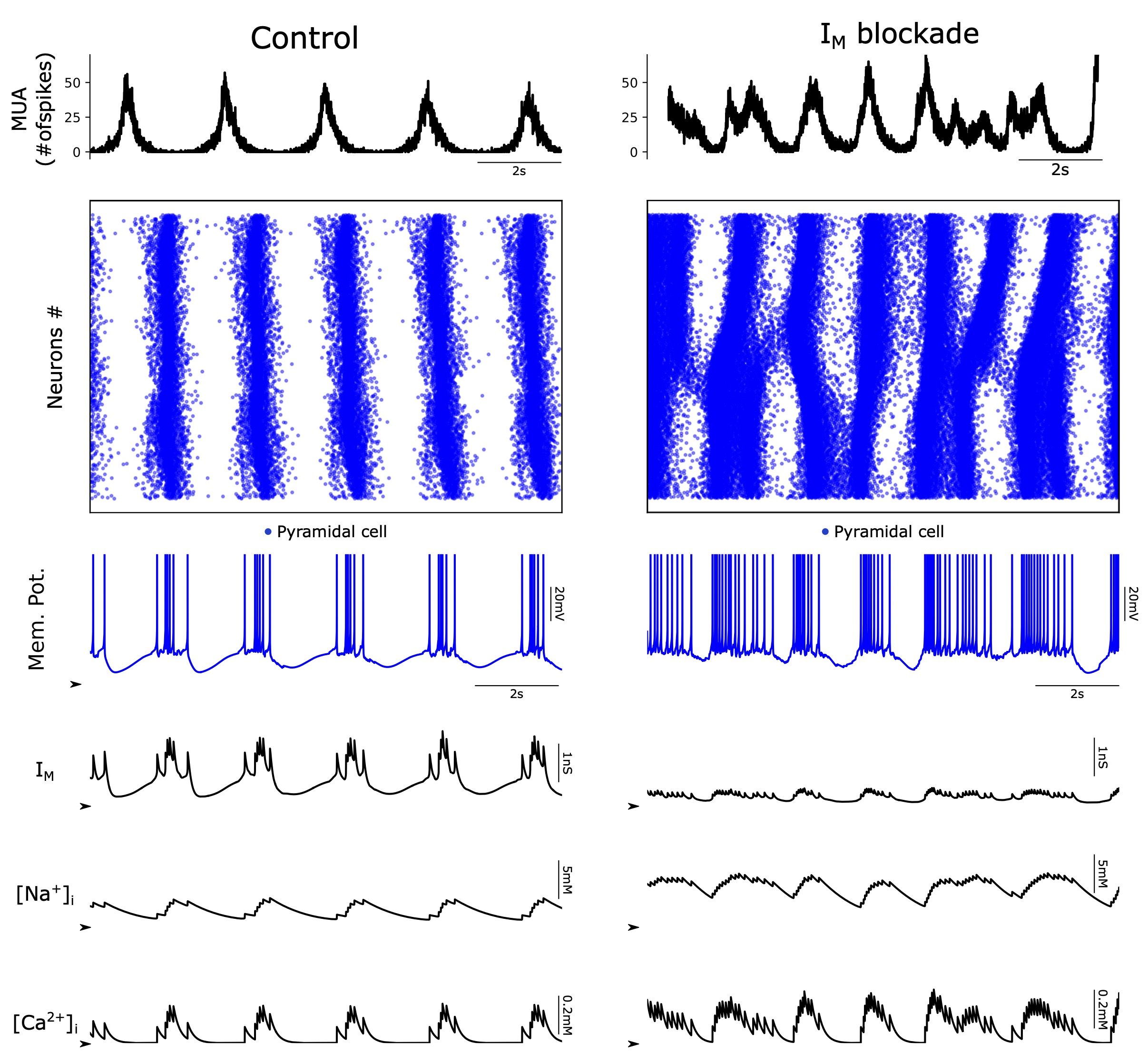

Supplement: S2 Fig — Higher excitability is achieved by a depolarizing current in all excitatory neurons (0.02 nA). Left column: control activity. Right column: M-current blockade (80%). (JPEG) [file pcbi.1011246.s002.jpeg]
